# Usefulness of sialic acid for diagnosis of sepsis in critically ill patients: a retrospective study

**DOI:** 10.1186/s12871-020-01197-2

**Published:** 2020-11-03

**Authors:** Bo Yao, Wen-juan Liu, Di Liu, Jin-yan Xing, Li-juan Zhang

**Affiliations:** 1 Systems Biology and Medicine Center, The affiliated hospital of Qingdao University, Wutaishan road 1677, Qingdao city, 26600 China; 2 The department of Critical Care Medicine, The affiliated hospital of Qingdao University, Wutaishan road 1677, Qingdao city, 26600 China

**Keywords:** Sepsis, Sialic acid, Procalcitonin, Virus, Bacteria, Corona virus disease 2019

## Abstract

**Background:**

Early diagnosis of sepsis is very important. It is necessary to find effective and adequate biomarkers in order to diagnose sepsis. In this study, we compared the value of sialic acid and procalcitonin for diagnosing sepsis.

**Methods:**

Newly admitted intensive care unit patients were enrolled from January 2019 to June 2019. We retrospectively collected patient data, including presence of sepsis or not, procalcitonin level and sialic acid level. Receiver operating characteristic curves for the ability of sialic acid, procalcitonin and combination of sialic acid and procalcitonin to diagnose sepsis were carried out.

**Results:**

A total of 644 patients were admitted to our department from January 2019 to June 2019. The incomplete data were found in 147 patients. Finally, 497 patients data were analyzed. The sensitivity, specificity and area under the curve for the diagnosis of sepsis with sialic acid, procalcitonin and combination of sialic acid and procalcitonin were 64.2, 78.3%, 0.763; 67.9, 84.0%, 0.816 and 75.2, 84.6%, 0.854. Moreover, sialic acid had good values for diagnosing septic patients with viral infection, with 87.5% sensitivity, 82.2% specificity, and 0.882 the area under the curve.

**Conclusions:**

Compared to procalcitonin, sialic acid had a lower diagnostic efficacy for diagnosing sepsis in critically ill patients. However, the combination of sialic acid and procalcitonin had a higher diagnostic efficacy for sepsis. Moreover, sialic acid had good value for diagnosing virus-induced sepsis.

## Background

Sepsis is common in the ICU. Approximately 35% of critically ill patients in the ICU meet the criteria for sepsis. In addition, the mortality of sepsis patients is high, approximately 33.1% [1]. The required timing for finishing sepsis bundles changes from 3 h to 1 h now [2]. This shows the importance of timing for sepsis treatment. For example, antimicrobial therapy is recommended in 1 h sepsis bundles. A delay in starting antimicrobial therapy is associated with high mortality [3]. Therefore, early sepsis diagnosis is very important. In the clinic, procalcitonin (PCT), as a biomarker, is widely used for the early diagnosis of sepsis. However, its pooled sensitivities and specificities were not higher than 0.80 in a recent meta-analysis study [4]. Therefore, it is necessary to find more effective and adequate biomarkers to diagnose sepsis.

Serum sialic acid (SA), as a common clinical biomarker, is used for cancer diagnosis. SA are typically found at the outermost ends of glycan chains in cells. In addition, sialic acid-binding immunoglobulin-type lectins are important receptors in immune cells [5]. Moreover, an uncontrolled host immune response to infection exists during sepsis [6]. Therefore, we speculated that serum SA levels could change during sepsis and have diagnostic value for sepsis. In this study, we compared the value of serum SA and PCT for diagnosing sepsis or different etiological sepsis (bacteria, fungi and virus).

## Patients and methods

This was a retrospective study. The study was approved by the Ethics Committee of the affiliated hospital of Qingdao University (No. QYFY WZLL 25945) and has therefore been performed in accordance with the ethical standards laid down in the 1964 Declaration of Helsinki and its later amendments. Newly admitted ICU patients were consecutively enrolled in this study from January 2019 to June 2019. From January 2020 to February 2020, we retrospectively collected patient data by electronic records, including primary disease, age, sex, Acute Physiology and Chronic Health Evaluation (APACHE) II scores, the presence of sepsis or not, and baseline (within 24 h from admission) serum SA and PCT values. The serum total sialic acid was measured by an enzymatic colorimetric assay (sialic acid, Dongou®, China). Sepsis diagnosis conformed to the sepsis 3.0 criterion [6].

In addition, we retrospectively collected SA level in 20 confirmed cases of Corona Virus Disease 2019 (COVID 2019) in our hospital from January 2020 to February 2020.

## Statistics analysis

The statistical analysis was performed using SPSS 22.0 software (SPSS, Inc., Chicago, IL, USA). In normal distribution quantitative data, the results were expressed as mean ± standard deviation. In Non normal distribution quantitative data, the results were expressed as median (quartile range). The prediction probability value of the combination of SA and PCT in sepsis was performed with binary logistic analysis. Receiver operating characteristic (ROC) curves for the ability of SA, PCT and prediction probability values to diagnose sepsis were carried out, and cut-off points were obtained from the curves for the highest sum of sensitivity and specificity. A value of P< 0.05 was considered statistically significant.

## Results

**Fig. 1:**
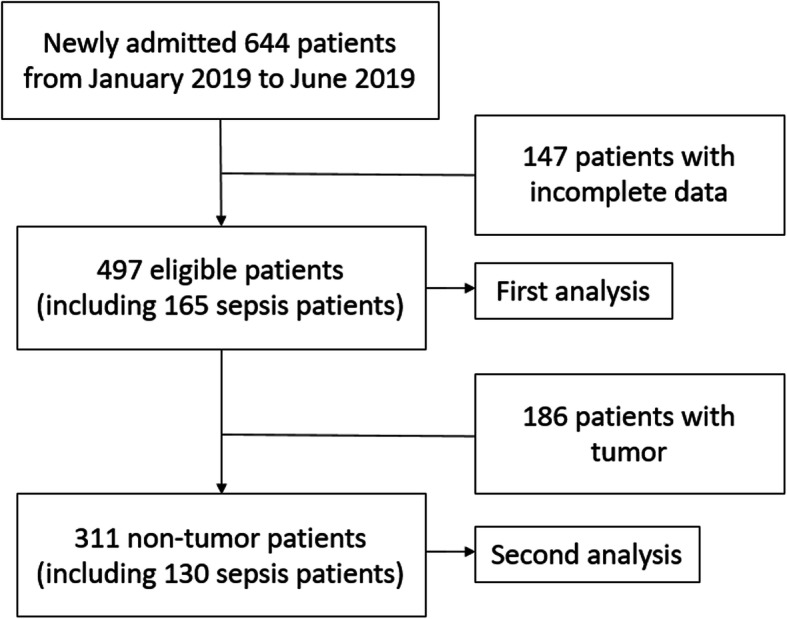
Flow chart

**Fig. 2:**
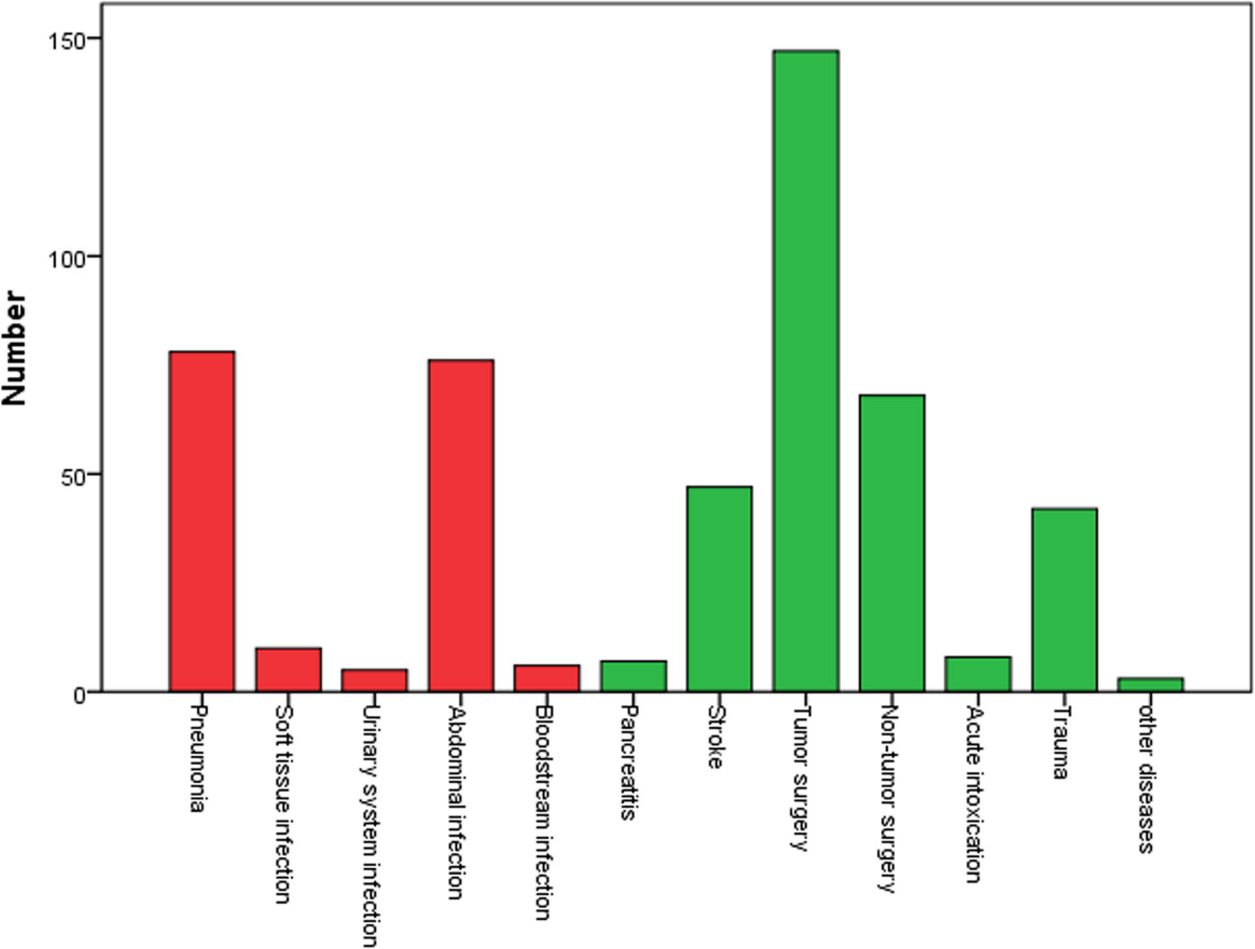
Diagnosis of 497 enrolled patients

A total of 644 patients were admitted to ICU from January 2019 to June 2019. The incomplete data was found in 147 patients (sepsis 4, tumor surgery 60, non-tumor surgery 67, stroke 11 and trauma 5). Finally, 497 patients data was analyzed (Fig. 1). In these 497 patients, 295 patients were male. APACHE II scores were 12(7–18). Age was 62 (50–72) years. The hospital mortality was 10.5%. 165 patients (33.2%) were diagnosed as sepsis. The main infection locations were lung (78), abdomen (76) and soft tissue (10). In non-infection patients, patients with high-risk surgery (215), stroke (47) and trauma (42) were common (Fig. 2).

**Fig. 3:**
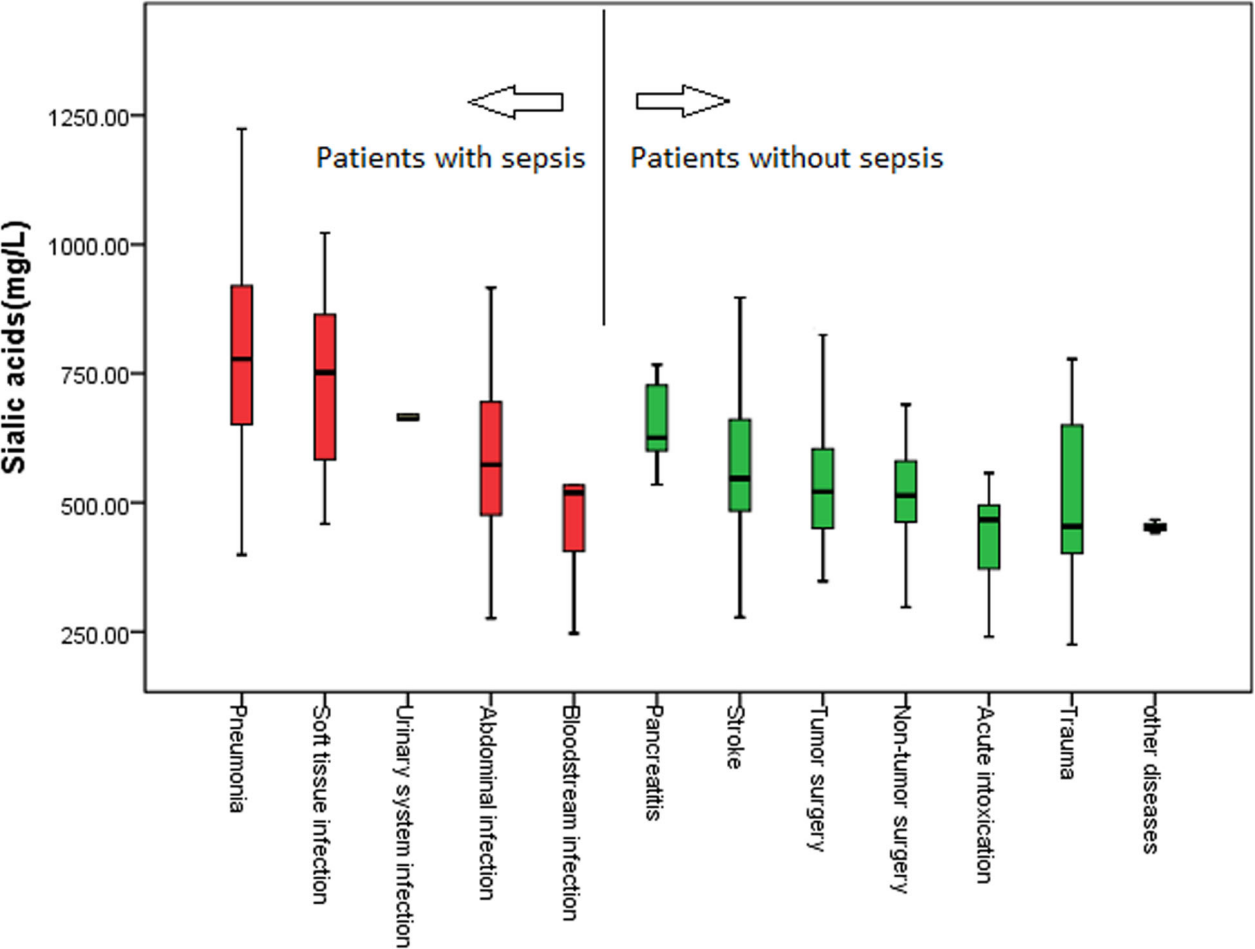
Sialic acid levels in different diseases

**Fig. 4:**
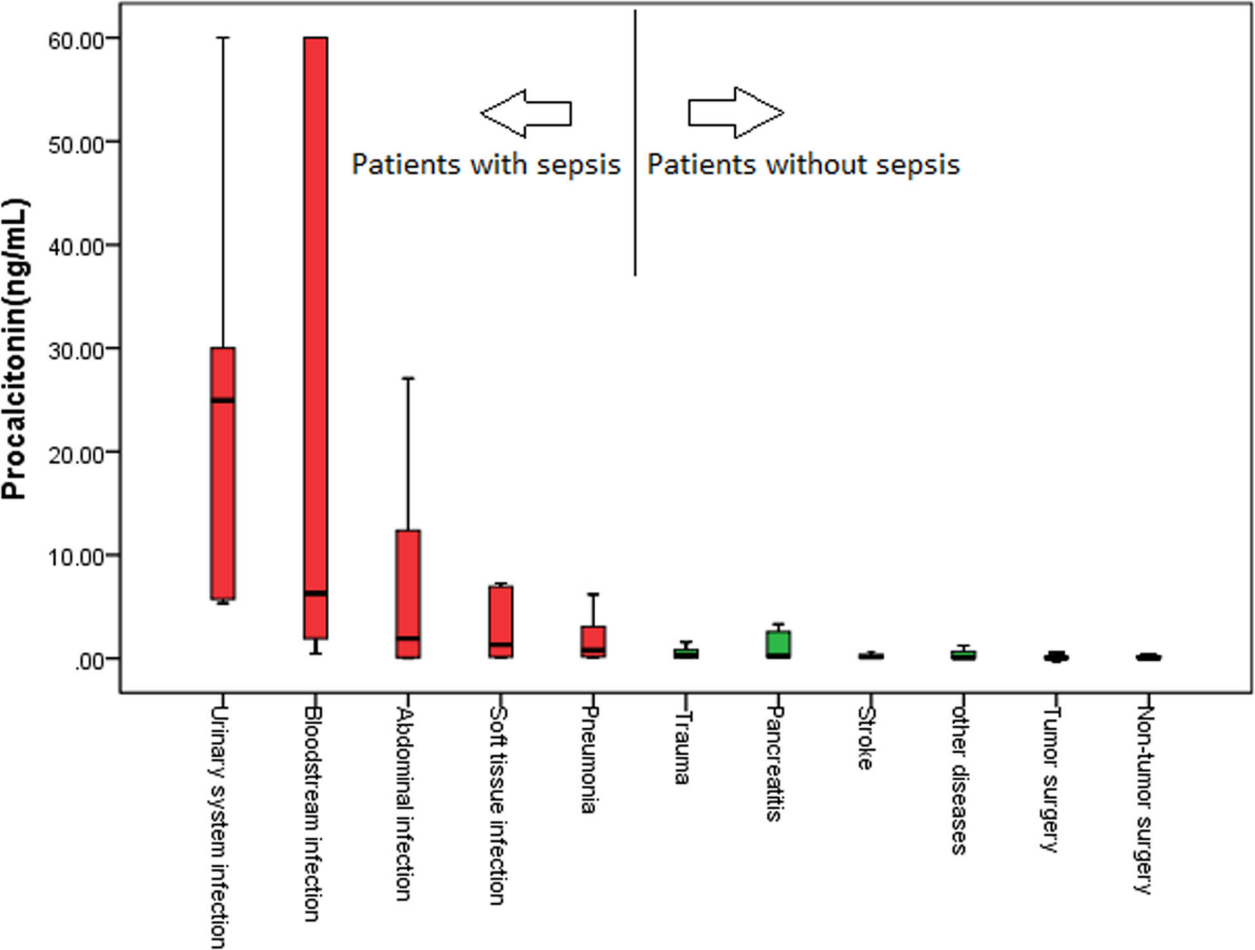
Procalcitonin levels in different diseases

The SA and PCT in septic patients were both higher than non-septic patients [689.6 (561.2–843.3) mg/L vs. 520.7 (451.0–604.3) mg/L, *P*<0.001; 1.67 (0.17–8.19) ng/mL vs. 0.05 (0.04–0.26) ng/mL, *P*<0.001] (Table 1). The disease with highest SA level in infection diseases was pneumonia [778.0 (651.2–922.6) mg/L], but the PCT level of pneumonia [0.80 (0.15–3.10) ng/mL] was lowest in infection diseases. The disease with lowest SA level in infection diseases was blood stream infection [519.4 (366.0–635.9) mg/L], but the PCT level of blood stream infection was second highest in infection diseases [6.30 (1.57–60.00) ng/mL]. The disease with highest SA level in non-infection diseases was pancreatitis [626.0 (591.8–766.6) mg/L] (Fig. 3). The disease with highest PCT level in non-infection diseases was trauma [0.29 (0.07–0.82) ng/mL] (Fig. 4).

**Table 1:** Baseline data in all enrolled patients

Items	Sepsis patients (*n* = 165)	Non-sepsis patients (*n* = 332)	*P* value
Male/Female	94/71	201/131	0.445
Age (years)	63 (53–72)	62 (48–72)	0.354
APACHEII scores	16 (11–24)	10 (6–15)	<0.001
PCT (ng/ml)	1.67 (0.17–8.19)	0.05 (0.04–0.26)	<0.001
SA (mg/L)	689.6 (561.2–843.3)	520.7 (451.0–604.3)	<0.001

*APACHE II* Acute Physiology and Chronic Health Evaluation, *PCT* procalcitonin, *SA* sialic acid

**Fig. 5:**
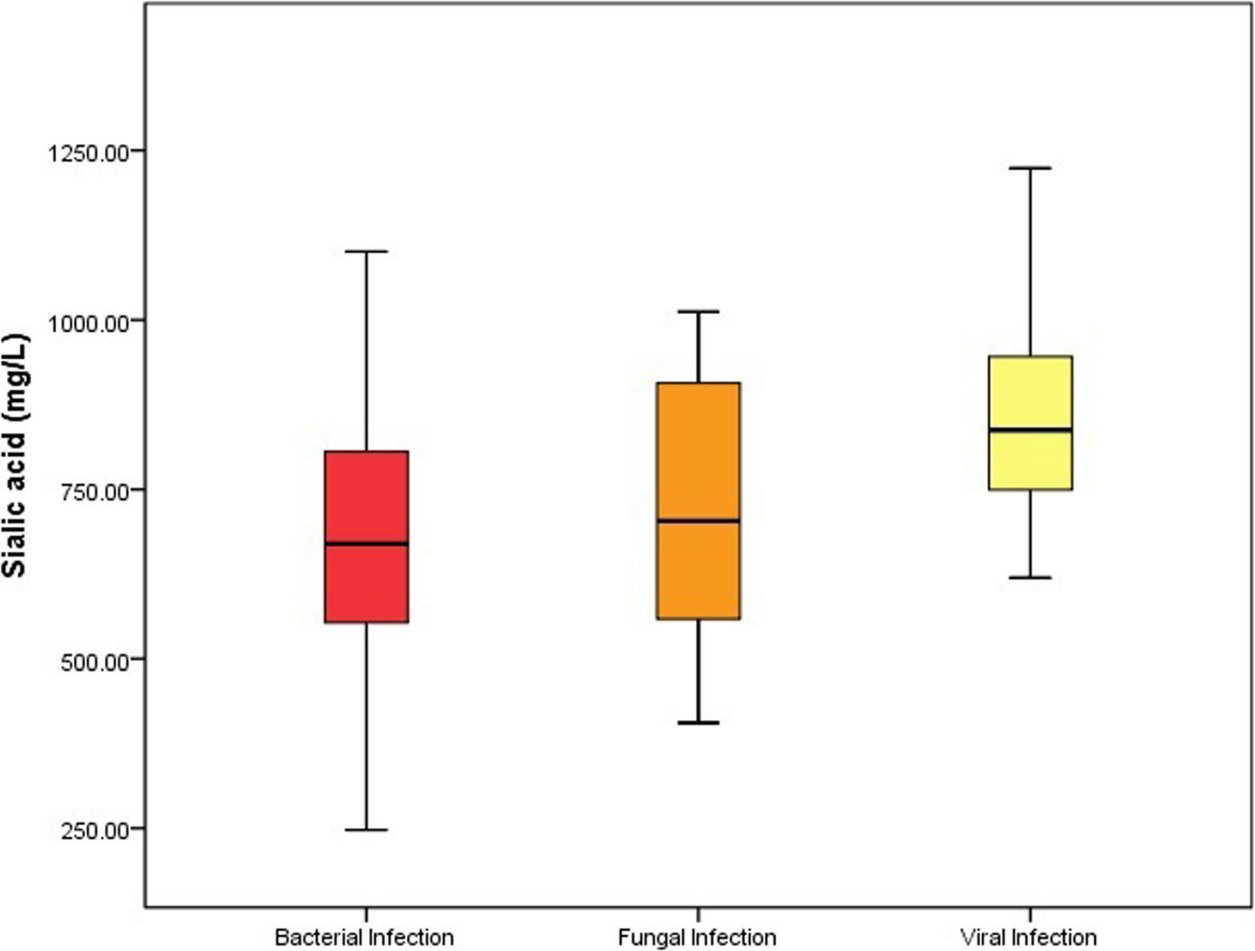
Sialic acid levels in different pathogens infection

In these 165 septic patients, 142 patients was infected with bacteria, 15 patients was infected with fungi and 8 patients was infected with virus. There was statistical difference of SA levels among bacterial infection, fungal infection and viral infection (684.3 ± 184.7 mg/L vs. 734.1 ± 214.0 mg/L vs. 863.5 ± 184.0 mg/L, *P* = 0.025). But there was no statistical significance in pairwise comparisons between any two of the three groups (P>0.05) (Fig. 5). In 20 patients with COVID 2019, 7 patients was diagnosed as sepsis. The SA level was much higher in septic patients with COVID 2019 than non-septic patients with COVID 2019 (804.5 ± 96.5 mg/L vs. 614.9 ± 117.7 mg/L, *P* = 0.002).

For all enrolled 497 patients, SA levels of 618.1 mg/L were diagnostic for sepsis with 64.2% sensitivity and 78.3% specificity, and the area under the curve was 0.763 (95% confidence interval 0.715–0.810). PCT levels of 0.435 ng/mL were diagnostic for sepsis with 67.9% sensitivity and 84.0% specificity, and the area under the curve was 0.816 (95% confidence interval 0.774–0.857). The logistic regression model by SA and PCT was logit (Probability) =0.245 × PCT + 0.007 × sialic acids - 5.769. Prediction probability value of 0.335 by SA and PCT were diagnostic for sepsis with 75.2% sensitivity and 84.6% specificity, and the area under the curve was 0.854 (95% confidence interval 0.816–0.893) (Table. 2). In addition, SA has good values for diagnosing septic patients with viral infection, with 87.5% sensitivity, 82.2% specificity, and 0.882 the area under the curve.

**Table 2:** The value of SA and PCT for diagnosing sepsis

Items	Cut-off value	Sensitivity (%)	Specificity (%)	Area under the curve	95% confidence interval
*For diagnosis of sepsis with all pathogen*					
SA (mg/L)	618.1	64.2	78.3	0.763	0.715–0.810
PCT (ng/ml)	0.435	67.9	84.0	0.816	0.774–0.857
Prediction probability value of SA and PCT	0.335	75.2	84.6	0.854	0.816–0.893
*For diagnosis of sepsis with bacterial infection*					
SA (mg/L)	571.3	73.2	64.5	0.726	0.675–0.778
PCT (ng/ml)	0.580	66.9	83.9	0.806	0.762–0.851
*For diagnosis of sepsis with viral infection*					
SA (mg/L)	727.9	87.5	82.2	0.885	0.810–0.960
PCT (ng/ml)	1.785	62.5	80.6	0.691	0.516–0.865
*For diagnosis of sepsis with fungal infection*					
SA (mg/L)	692.5	0.667	77.2	0.702	0.543–0.861
PCT (ng/ml)	0.055	93.3	38.0	0.654	0.537–0.770
*For diagnosis of sepsis in non-tumor patients*					
SA (mg/L)	571.3	75.4%	70.7%	0.774	0.719–0.828
PCT (ng/ml)	0.58	63.1%	81.8%	0.771	0.718–0.825
Prediction probability value of SA and PCT	0.453	72.3%	85.1%	0.843	0.797–0.890

*PCT* procalcitonin, *SA* sialic acid

In these 497 patients, 186 patients (37.4%) suffered from tumor. Most of them were patients with high-risk surgery. The SA in tumor patients was lower than non-tumor patients (544.7[454.3–646.4] mg/L vs. 563.8[469.5–708.0] mg/L, *P* = 0.022). We further analyzed this 311 non-tumor patients data (Fig. 1). SA levels of 571.3 mg/L were diagnostic for sepsis with 75.4% sensitivity and 70.7% specificity, and the area under the curve was 0.774 (95% confidence interval 0.719–0.828). PCT levels of 0.58 ng/ml were diagnostic for sepsis with 63.1% sensitivity and 81.8% specificity, and the area under the curve was 0.771 (95% confidence interval 0.718–0.825). The logistic regression model by SA and PCT was logit (Probability) = 0.180 × PCT + 0.007 × sialic acids - 5.354. Prediction probability value of 0.453 were diagnostic for sepsis with 72.3% sensitivity and 85.1% specificity, and the area under the curve was 0.843 (95% confidence interval 0.797–0.890) (Table. 2).

For all enrolled 497 patients, the area under the curve of Prediction probability, SA and PCT to predict the hospital mortality was 0.670, 0.620 and 0.710. For these 311 patients without tumor, the area under the curve of Prediction probability, SA and PCT to predict the hospital mortality was 0.612, 0.583 and 0.662.

## Discussion

Despite recent developments in the treatment of sepsis, morbidity and mortality remain high [1]. Early diagnosis of sepsis can improve the clinical outcomes of sepsis [7]. Although pathogen detection remains the gold standard for diagnosing infection, positive blood cultures account for only 30–40% of sepsis cases [8]. In addition, the presence of pathogens does not confirm the existence of infections. It is possible that bacterial colonization is present. Blood cultures also take too much time and provide limited help for early diagnosis. PCT is a widely used biomarker in sepsis. PCT is the inactive propeptide of calcitonin, which is mainly released by C cells of the thyroid gland [9]. The pooled area under the ROC curve of PCT for sepsis diagnosis was 0.84 [4]. In our study, the area under the ROC curve of PCT for diagnosing sepsis also had similar values. However, PCT may only slightly increase in patients with sepsis caused by fungi infection or virus infection [10]. In addition, PCT can also increase in some diseases in the absence of infection, such as trauma. Some studies have also produced conflicting results regarding its diagnostic value [11–14]. Only a weak recommendation was provided about PCT in the 2016 Surviving Sepsis Campaign guidelines [7]. Therefore, it is necessary to find more effective biomarkers to discriminate sepsis from noninfectious critical illnesses.

SA are typically found at the outermost ends of glycan chains in all cell types. Furthermore, SA has three important biological and pathological roles. First, because of its negative charge and hydrophilicity, SA plays a role in blood cell charge repulsion. Second, some pathogens can recognize and bind SA on the cell membrane, and SA acts as a ligand for extrinsic receptors. Third, SA also serves as a ligand for intrinsic receptors such as sialic acid-binding immunoglobulin-type lectins (Siglecs). Siglecs containing 2–17 extracellular Ig domains are found on the surface of innate and adaptive immune cells [15]. Sepsis is defined as life-threatening organ dysfunction induced by an uncontrolled host response to invading pathogens. Dysregulation of immune function by immune cells is characteristic of sepsis. As important receptors in immune cells, siglecs are involved in the pathogenesis of sepsis [5]. Serum SA is an easily obtained clinical laboratory index. Huang X et al. found that patients with sepsis had the highest mean serum SA levels among 64 diseases [16]. However, this study only showed that the level of SA increased in patients with sepsis compared to the level in healthy individuals. In our study, we further examined the role of SA in diagnosing sepsis in ICU patients. Our study showed that the SA level in sepsis patients was higher than that in non-sepsis patients. However, we did not find that SA was a better biomarker than PCT for diagnosing sepsis in critically ill patients. But we found that SA level was highest in viral infection. And SA had good value for diagnosing sepsis with viral infection. In COVID 2019 patients, the SA level was also higher in sepsis than non-sepsis. It was confirmed that SA was critical for virus infection process [17]. PCT usually increased highly in bacterial infection other than virus infection. Chalupa P et al. confirmed that PCT could discriminate between bacterial and viral infections (mainly Tick-borne encephalitis and Enteroviral meningitis) [18]. But in our study the area under the curve of PCT for diagnosing virus-induced sepsis was only 0.691. The reason may be that some virus-induced sepsis in our study included mixed infection by virus and bacteria. Moreover, the enrolled patients were all ICU patients (including critically ill patients without infection) in our study, but the enrolled patients in the study by Chalupa P et al. were only infected patients in standard wards of the department of Infectious Diseases. Now, there was few very good biomarkers to distinguish between virus infection and other types of infection [19]. In our study, SA was shown as a good biomarker for diagnosing virus-induced sepsis in critically ill patients. Because of small sample, further large sample study is needed to confirm the result.

A general upregulation of sialylated glycans on cell surfaces occurs in the tumor microenvironment [20]. The glycoproteins and glycolipids expressed by tumors can be released into the serum through some pathways [21]. Therefore, serum SA levels have usually been tested routinely in hospitals for cancer monitoring. We speculated that tumor may be a confounding factor for sepsis diagnosis by SA. Interestingly, in critically ill non-tumor patients, SA had a higher sensitivity and a slightly larger area under the ROC curve than PCT for diagnosing sepsis. In addition, the PCT diagnostic value for sepsis decreased in critically ill non-tumor patients compared to all critically ill patients. In critically ill non-tumor patients, the proportion of patients with trauma increased from 8.5 to 13.5%. A previous study confirmed that PCT levels can increase to 1.8 ng/ml after 24 h of trauma [22]. In our study, patients with trauma has the highest PCT level in all non-infection diseases. Therefore, the increasing proportion of patients with trauma may decrease the diagnostic value of PCT for sepsis in non-tumor patients. Moreover, we found that SA in tumor patients was lower than non-tumor patients. So SA is not a good biomarker for cancer monitoring for critically ill patients.

Specificity is important for the diagnosis. A diagnostic biomarker with a low specificity can diminish the improper use of antibiotics. In a cohort study, among 2579 patients treated for sepsis, approximately half of the patients had a post hoc likelihood of infection of none or only possible [23]. Patients with an unlikely infection but who were initially treated for sepsis have a higher mortality rate than patients with a definite infection [23]. This study implied that the diagnosis of sepsis in critically ill patients was likely overestimated. Antibiotic therapy is likely excessive treatment. Therefore, a high-specificity biomarker is important for diagnosing sepsis. We found that the specificity of SA for diagnosing sepsis was nearly 80% in our study. But sensitivity is also important for the diagnosis of sepsis. A low sensitivity can result in a high missed rate. It is dangerous to misdiagnose sepsis patients because of the high mortality associated with sepsis. In this study, SA had a low sensitivity in all critically ill patients. However, our results showed that in some diseases, such as pneumonia and blood stream infection, the SA and PCT has contrary level change. So the combination of SA and PCT may has more diagnostic value. Further, we found that the combined application of SA and PCT had a higher sensitivity, specificity and area under ROC curve than the use of SA or PCT alone.

In this study, SA in sepsis patients was higher than no-sepsis patients. We further analyze the SA levels in different infection locations. And finally we found that SA level was different in different infections. SA level was much higher in pneumonia than blood stream infection. But PCT has contrary level change. Moreover, in different etiological types of sepsis, SA levels was highest in viral sepsis. The reasons behind these phenomena needed further studies to elucidate it.

There were some limits in this study. Because it is a retrospective study, and more than 20% patient data were incomplete. The missing data were excluded from the analysis, so the efficiency of this diagnostic study decreased. In addition, we only collected the SA data in ICU admission. The value of dynamic change of SA was not evaluated. Thirdly, because of small sample size in viral infection, further study is needed to confirm the value of SA for diagnosing sepsis with viral infection.

## Conclusions

Compared to PCT, SA was not a good biomarker for diagnosing sepsis in critically ill patients. However, the combination of SA and PCT had a higher diagnostic efficacy for sepsis. In addition, SA had good value for diagnosing sepsis with viral infection. And sialic acid level was much higher in septic patients with COVID 2019 than non-septic patients with COVID 2019. Both SA and PCT were not good biomarkers to predict the hospital mortality.

## Data Availability

The datasets during the current study available from the corresponding author on reasonable request.

## Abbreviations

PCT: Procalcitonin
SA: Sialic acid
APACHE: Acute Physiology and Chronic Health Evaluation
COVID 2019: Corona Virus Disease 2019
ROC: Receiver operating characteristic
